# Antibiotic prophylaxis—Preventing severe infections and saving lives in poor countries with very high mortality risk

**DOI:** 10.1371/journal.pmed.1002594

**Published:** 2018-06-26

**Authors:** Keith P. Klugman, Rasa Izadnegahdar

**Affiliations:** 1 Pneumonia Program Strategy Team, Bill and Melinda Gates Foundation, Seattle, Washington, United States of America; 2 Maternal Neonatal, Child Health Discovery and Tools Program Strategy Team, Bill and Melinda Gates Foundation, Seattle, Washington, United States of America

## Abstract

In a Perspective, Keith P. Klugman and Rasa Izadnegahdar from the Bill and Melinda Gates Foundation discuss the potentials and risks of antibiotic prophylaxis interventions for infectious disease outbreaks in rural regions, such as sub-Saharan Africa with very high mortality rates, when primary prophylactic vaccination programs are not yet available.

Improvements in child survival are among the most significant achievements in global health during the 21st century. More progress is required, however, to reach the mortality targets established by the Sustainable Development Goals (SDGs). Compared to the average 2%–4% annual reduction in mortality rate for children under 5 years of age (U5MR) observed in sub-Saharan Africa during the Millennium Development Goal (MDG) era, a substantial acceleration to 6%–9% reduction per year will be required to attain these 2030 targets [1]. This kind of progress requires strategic consideration of the evidence for existing tools, such as antibiotic prophylaxis, to reduce deaths in very high mortality settings (U5MR greater than 90 per 1,000 live births), which are disproportionately affected by infectious diseases [2]. The top 10 of these are African countries—including Chad, Somalia, and Mali in the meningitis belt—and they include 3 large countries with the largest number of under–5-year-old deaths (Angola, Nigeria, and Democratic Republic of the Congo) [2]. There are additional countries with geographic areas of mortality in excess of 90/1,000 live births, such as Niger, where 2 studies of mass antibiotic prophylaxis have recently been published [3,4].

Niger is in the meningitis belt just south of the Sahara Desert, where, in addition to very high infant mortality, the population is afflicted by meningitis epidemics, during which up to 1% of the population may be affected [5]. Campaigns of a conjugated meningococcal group A vaccine have substantially prevented both meningococcal disease and its transmission for a number of years [6]. In high-income countries, targeted vaccination is supplemented by antibiotic prophylaxis among household contacts of cases of nonepidemic meningococcal meningitis. [7]. The WHO position for antibiotic prophylaxis in the setting of meningitis epidemics is that “antibiotics are recommended as a prophylactic measure for household contacts of all ages in nonepidemic periods but not during epidemics.” The guideline summarizes current data as follows:

Antibiotic prophylaxis is currently recommended for household contacts of those with sporadic invasive meningococcal disease, but not for widespread use or administration to household contacts during epidemics. Thus, before an epidemic is declared, this policy should be applied for affected families. The consensus of the Guideline Development Group was that, whereas the benefit of antibiotic prophylaxis in the meningitis belt is uncertain, the cost of giving single-dose ciprofloxacin to household contacts and the risk of adverse effects are both low, such that benefit may outweigh harm even if the absolute benefit is small. However, the group did not find any new information to suggest that, during epidemics, chemoprophylaxis for household contacts of cases would offer any additional benefit to the community in situations where case management and vaccination programs are being implemented. For vaccination as a household prophylactic measure ahead of mass vaccination, the group considered that any additional benefit of prophylaxis ahead of mass vaccination would be small in relation to the difficulties of implementation, particularly where mass vaccination is planned for the community.

The WHO document does, however, conclude that “the effectiveness of this intervention would be best evaluated in a randomized trial. However, the logistic difficulties of mounting a trial across districts and countries, with a sufficient sample size of cases in household contacts outside epidemics, may be prohibitively large” [7].

## Ciprofloxacin single-dose prophylaxis prevents meningitis during an epidemic

A cluster-randomized trial of this scale funded by Epicentre and Medicine Sans Frontieres is reported by Matthew Coldiron and colleagues in this issue of *PLOS Medicine*, in a setting in which an epidemic had been declared but provision of vaccine was delayed [4]. They randomized villages in Niger during a meningococcal group C epidemic in 2017 to receive either the current WHO standard of care of no antibiotic prophylaxis, household prophylaxis with a single dose of ciprofloxacin, or village-wide (mass) single-dose ciprofloxacin prophylaxis within 72 hours of the notification of the first meningitis case in a village. Household prophylaxis reduced the attack rate of subsequent cases by a nonsignificant 6%, suggesting that transmission of meningococci in remote rural villages may be more village-wide than household-specific. Indeed, mass prophylaxis with a mean ciprofloxacin coverage of 77% (range 56%–100%) led to an 82% (95% CI 67%‒90%; *P* < 0.0001) reduction in secondary cases among those people in all study villages who actually received the drug. The overall reduction in secondary meningitis cases in the villages receiving mass prophylaxis compared to control villages was 60% (95% CI 13%–81%; *P* = 0.022).

While the benefit of mass prophylaxis is clear in this study, it must be balanced against its impact on resistance. Ciprofloxacin-resistant pneumococci have been isolated globally but remain rare and largely sporadic [8]. Ciprofloxacin-resistant meningococcal strains were not directly measured in the study; however, the carriage rate of ciprofloxacin-resistant Enterobacteriaceae in stool in this remote rural African setting was noted to be as high as 95%, with the carriage of extended-spectrum–resistant Enterobacteriaceae similarly >90%. Both percentages for resistance remained unchanged following the intervention.

In light of these data, WHO may consider revising its recommendations to include consideration of targeted mass provision of ciprofloxacin in high–meningitis-burden settings, at least until individuals are vaccinated or the epidemic ends.

## Single-dose azithromycin prophylaxis prevents mortality

Niger is not often the epicenter of 2 major infectious disease trials—let alone 2 antibiotic prophylaxis studies—but in an unrelated study, a single twice-yearly dose of azithromycin given prophylactically within randomized villages to children less than 5 years of age in districts of Niger, Tanzania, and Malawi led to an 18% overall reduction in all-cause mortality in Niger compared to control villages (95% CI 10%–25.5%; *P* < 0.001). In subgroup analysis by age, the highest level of impact was in children 1 to 5 months of age (24.9% mortality reduction overall; 95% CI 10.6%–37.0%; *P* = 0.001) [3]. Of particular interest for the mechanism of action was the observation that the efficacy did not decrease over the 4 rounds of mass drug administration, and there was a nonsignificant increase in efficacy over the 2 years of follow-up. If community-wide azithromycin exposure is protecting these children by altering their microbiome, it is possible that the effect could increase with each subsequent round due to a sharing of the favorable microbiome among untreated children in the villages.

Azithromycin given as part of trachoma mass drug administration to all community members over 6 months of age leads to selection of macrolide-resistant pneumococci, the magnitude of which is proportional to pre–mass drug administration levels of macrolide-resistant clones [8]. This drug is fortunately not used widely for childhood pneumonia treatment in Africa. Nonetheless, if the mortality prevention effects are mediated by the antibacterial action—even if this is indirect by allowing colonization with less virulent competing strains—the risk that the effect will be lost over time, owing to the emergence of resistance, is real.

## The way forward

These 2 trials of village-wide antibiotic prophylaxis suggest a major role for targeted mass single-dose antibiotic prophylaxis programs, at least in the short term. Such a strategy would be beneficial in high-mortality, high–disease-burden settings such as Niger until a multivalent conjugate vaccine that is protective beyond serogroup A meningitis is available and U5MR is reduced to lower than 90 deaths per 1,000 live births.

Single-dose antimicrobial prophylaxis may have profound effects in the meningitis belt, which includes some of the poorest countries in sub-Saharan Africa—the long-term cost-to-benefit ratio in these communities should determine the way the antimicrobials are used.

## Abbreviations

MDG: Millennium Development Goal
SDG: Sustainable Development Goal
U5MR: mortality rate for children under 5 years of age

## References

[1] Golding N, Burstein R, Longbottom J, et al Mapping under-5 and neonatal mortality in Africa, 2000–15: a baseline analysis for the Sustainable Development Goals. Lancet. 2017 11 11;390(10108):2171–2182. doi: 10.1016/S0140-6736(17)31758-0 28958464 10.1016/S0140-6736(17)31758-0 PMC5687451

[2] Liu L, Oza S, Hogan D, Chu Y, Perin J, Zhu J, et al Global, regional, and national causes of under-5 mortality in 2000–15: an updated systematic analysis with implications for the Sustainable Development Goals. Lancet. 2016 12 17;388(10063):3027–3035. doi: 10.1016/S0140-6736(16)31593-8 27839855 10.1016/S0140-6736(16)31593-8 PMC5161777

[3] Keenan JD, Bailey RL, West SK, Arzika AM, Hart J, Weaver J, et al Azithromycin to Reduce Childhood Mortality in Sub-Saharan Africa. The New England journal of medicine. 2018;378(17):1583–92. doi: 10.1056/NEJMoa1715474 29694816 10.1056/NEJMoa1715474 PMC5849140

[4] Coldiron ME, Assao B, Page A-L, Hitchings MDT, Alcoba G, Ciglenecki I, et al Single-dose oral ciprofloxacin prophylaxis as a response to a meningococcal meningitis epidemic in the African meningitis belt: a three-arm, open-label, cluster-randomized trial. PLoS Med. 2018. 10.1371/journal.pmed.1002593 PMC6019097 29944651

[5] Lingani C, Bergeron-Caron C, Stuart JM, Fernandez K, Djingarey MH, Ronveaux O, et al Meningococcal Meningitis Surveillance in the African Meningitis Belt, 2004–2013. Clinical Infectious Diseases. 2015 11 15; 61(Suppl 5): S410–S415. 26553668 10.1093/cid/civ597 PMC4639499

[6] Daugla DM, Gami JP, Gamougam K, Naibei N, Mbainadji L, Narbe M, et al Effect of a serogroup A meningococcal conjugate vaccine (PsA-TT) on serogroup A meningococcal meningitis and carriage in Chad: a community study. Lancet. 2014 1 4;383(9911):40–47. doi: 10.1016/S0140-6736(13)61612-8 24035220 10.1016/S0140-6736(13)61612-8 PMC3898950

[7] Meningitis Outbreak Response in Sub-Saharan Africa: WHO Guideline. Available from: http://www.ncbi.nlm.nih.gov/books/NBK274193/. [cited 1 May 2018]. 25674656

[8] Harcourt BH, Anderson RD, Wu HM, Cohn AC, MacNeil JR, Taylor TH, et al Population-based surveillance of *Neisseria meningitidis* antimicrobial resistance in the United States. Open Forum Infect Dis. 2015 8 13;2(3):ofv117 doi: 10.1093/ofid/ofv117 26357666 10.1093/ofid/ofv117 PMC4561371

[9] Keenan JD, Klugman KP, McGee L, Vidal JE, Chochua S, Hawkins P, et al Evidence for clonal expansion after antibiotic selection pressure: pneumococcal multilocus sequence types before and after mass azithromycin treatments. J Infect Dis. 2015 3 15;211(6):988–94. doi: 10.1093/infdis/jiu552 25293366 10.1093/infdis/jiu552 PMC4416126

